# The Hsp90 inhibitor SNX-7081 is synergistic with fludarabine nucleoside via DNA damage and repair mechanisms in human, p53-negative chronic lymphocytic leukemia

**DOI:** 10.18632/oncotarget.5715

**Published:** 2015-11-06

**Authors:** Kimberley L. Kaufman, Yiping Jenkins, Munther Alomari, Mehdi Mirzaei, O. Giles Best, Dana Pascovici, Swetlana Mactier, Stephen P. Mulligan, Paul A. Haynes, Richard I. Christopherson

**Affiliations:** ^1^ School of Molecular Bioscience, University of Sydney, Darlington, NSW 2006, Australia; ^2^ Molecular Neuropathology, Brain and Mind Centre, Camperdown, NSW 2050, Australia; ^3^ Department of Chemistry and Biomolecular Sciences, Macquarie University, North Ryde, NSW 2109, Australia; ^4^ Northern Blood Research Centre, Kolling Institute for Medical Research, Royal North Shore Hospital, St Leonards, NSW 2065, Australia; ^5^ Australian Proteome Analysis Facility, Macquarie University, North Ryde, NSW 2109, Australia

**Keywords:** chronic lymphocytic leukemia (CLL), fludarabine nucleoside, Hsp90 inhibitor, MYC, NFkB2

## Abstract

Clinical trials of heat shock protein 90 (Hsp90) inhibitors have been limited by high toxicity. We previously showed that the Hsp90 inhibitor, SNX-7081, synergizes with and restores sensitivity to fludarabine nucleoside (2-FaraA) in human chronic lymphocytic leukemia (CLL) cells with lesions in the p53 pathway (Best OG, *et al*., Leukemia Lymphoma 53:1367-75, 2012). Here, we used label-free quantitative shotgun proteomics and comprehensive bioinformatic analysis to determine the mechanism of this synergy. We propose that 2-FaraA-induced DNA damage is compounded by SNX-7081-mediated inhibition of DNA repair, resulting in enhanced induction of apoptosis. DNA damage responses are impaired in part due to reductions in checkpoint regulators BRCA1 and cyclin D1, and cell death is triggered following reductions of MYC and nucleolin and an accumulation of apoptosis-inducing NF*k*B2 p100 subunit. Loss of nucleolin can activate Fas-mediated apoptosis, leading to the increase of pro-apoptotic proteins (BID, fas-associated factor-2) and subsequent apoptosis of p53-negative, 2-FaraA refractory CLL cells. A significant induction of DNA damage, indicated by increases in DNA damage marker ϕH2AX, was observed following the dual drug treatment of additional cell lines, indicating that a similar mechanism may operate in other p53-mutated human B-lymphoid cancers. These results provide valuable insight into the synergistic mechanism between SNX-7081 and 2-FaraA that may provide an alternative treatment for CLL patients with p53 mutations, for whom therapeutic options are currently limited. Moreover, this drug combination reduces the effective dose of the Hsp90 inhibitor and may therefore alleviate any toxicity encountered.

## INTRODUCTION

Long term follow up of clinical trials of fludarabine (2-FaraAMP) showed that drug resistance due to mutations in the p53 pathway represents a significant challenge in the clinical management of CLL patients [1]. 2-FaraAMP is dephosphorylated by 5′-nucleotidase at the cell surface to fludarabine nucleoside (2-FaraA), which enters the cell via several nucleoside-specific membrane transporters, and is converted to the cytotoxic triphosphate derivative (2-FaraATP). 2-FaraATP is incorporated into elongating DNA chains and terminates chain synthesis causing double-strand breaks (DSBs) [2]. The cellular response to DNA damage involves networks of proteins that induce either apoptosis or DNA repair; p53 plays a pivotal role in mediating cell fate decisions [3]. In cells with functional p53, DNA damage causes p53 phosphorylation and accumulation, inducing cell cycle arrest, down-regulation of DNA repair proteins [4, 5] and apoptosis, thereby preventing the proliferation of damaged cells [6]. Cells with mutated p53 (e.g., MEC1) are generally resistant to DNA damaging agents such as 2-FaraA [7, 8], due to an inability to down-regulate DNA repair proteins and induce apoptosis [9].

Previous work from our laboratory has shown that the Hsp90 inhibitor SNX-7081 synergizes with and restores sensitivity to 2-FaraA in CLL cells with lesions in the p53 pathway [10]. Hsp90 is a chaperone that ensures the correct folding and stability of more than 200 ‘client’ proteins, many of which are oncoproteins [11–13]. There have been more than 40 clinical trials of Hsp90 inhibitors against a range of cancers [14]. SNX-7081, a novel Hsp90 inhibitor developed by Serenex (now Pfizer, New York, NY, USA) is more potent and soluble than geldanamycin-derivative inhibitors such as 17-AAG [15]. Our previous study showed that SNX-7081 can induce apoptosis in p53-negative MEC1 CLL cells by deregulating proteins involved with DNA repair and replication, and the cell cycle [16]. However, despite the promise of Hsp90 inhibitors as anti-cancer agents, toxicity has limited their use [17]. Synergistic combinations of Hsp90 inhibitors with other chemotherapeutics can reduce the effective dose of the Hsp90 inhibitor required, thereby alleviating the toxicity. Our previous results showed that the Hsp90 inhibitor SNX-7081 has synergistic effects with 2-FaraA against the p53 mutant cell lines MEC1, MEC2 and U266, and 23 clinical samples of CLL [10]. In this paper, we have employed comprehensive quantitative shotgun proteomics to determine protein changes in MEC1 cells induced by 2-FaraA and SNX-7081 treatments alone and in combination. Extensive bioinformatics analysis predicted changes in the activation states of up-stream regulators that were then tested by immunoblotting. The results herein provide an explanation for this drug synergy.

## RESULTS

In total, 1,596 non-redundant proteins were identified in whole cell lysates of control and drug-treated human MEC1 cells. The numbers of proteins confidently identified were 1,050 in control cells, 1,163 following 2-FaraA treatment, 1,076 following SNX-7081 treatment and 1,221 following dual 2-FaraA+SNX-7081 treatment. Variation in numbers of identified proteins between the triplicate analyses was minimal and the number of peptides in each LC-MS/MS run was consistent between the treatments; numbers of identified proteins and peptides in each replicate, as well as the coefficient of variation are provided in Table 1. Calculated levels of peptide and protein false discovery rates were less than 0.1%.

**Table 1:** Number of identified proteins and peptides in MEC1 samples

Condition	Number of identified proteins			CV%^b^	Redundant peptide count			CV%^c^	No. of proteins identified in all 3 replicates
	R1^a^	R2	R3		R1^a^	R2	R3		
Control	1637	1907	1791	0.0762	39609	34407	33224	0.0950	1050
SNX-7081-treated	1769	1626	1772	0.0484	35223	35651	34067	0.0234	1076
2-FaraA treated	1811	2178	1840	0.1050	35704	38078	34300	0.0530	1163
2-FaraA + SNX-7081 treated	1843	1965	2211	0.0934	35410	38181	41135	0.0749	1221

a *R1, R2 and R3 denote replicate 1, replicate 2 and replicate 3, respectively*.

b *CV% denotescoefficient of variation of protein numbers between replicates*,

c *CV% is coefficient of variation of peptide numbers between replicates*.

**Figure 1:**
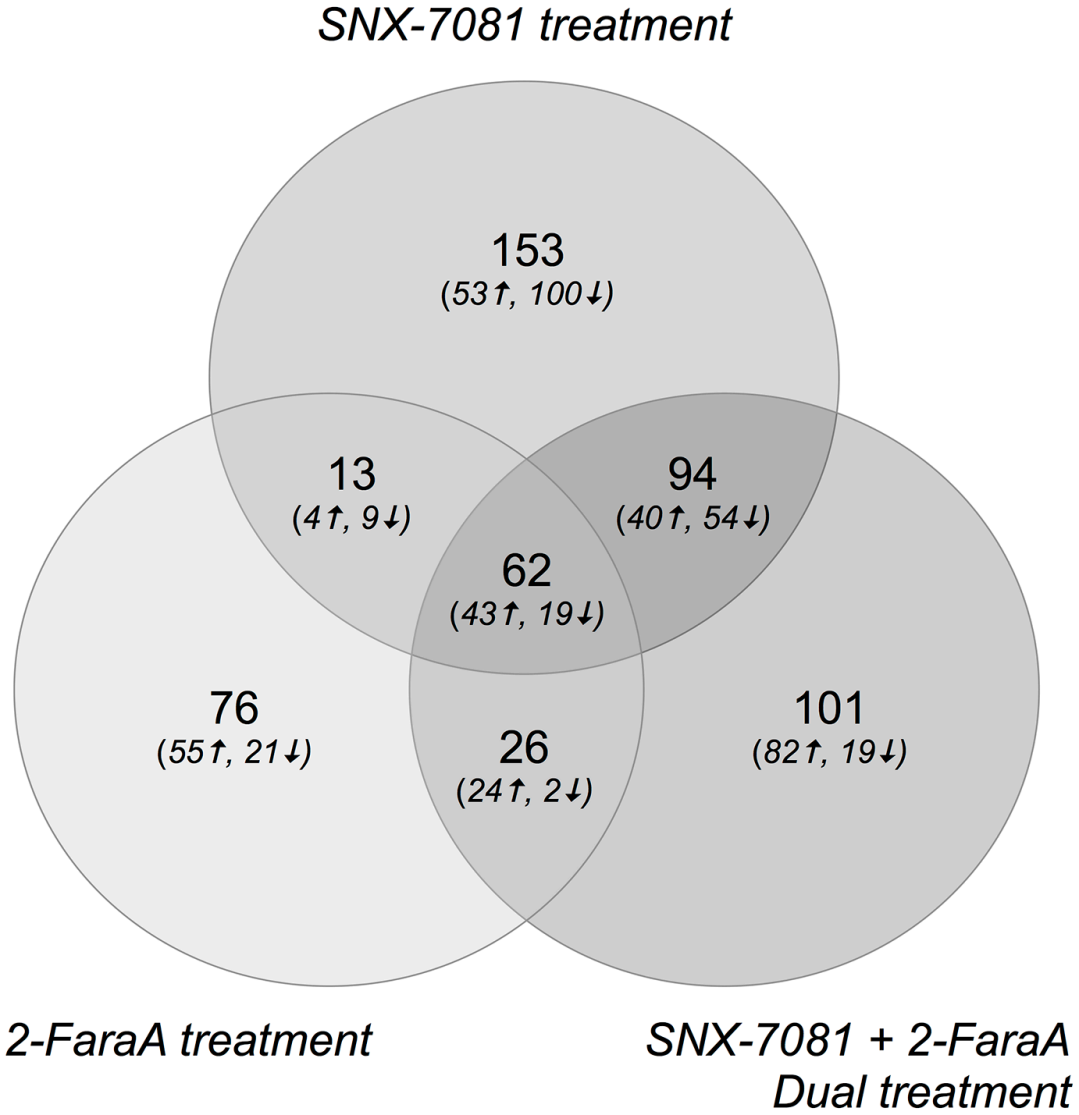
Venn diagram showing numbers of identified proteins that increased or decreased by more than 2-ratio (*p* < 0.05) after SNX-7081 (100 nM, 48 h), 2-FaraA (10 μM, 48 h) and SNX-7081 (100 nM) + 2-FaraA (10 μM) (48 h)

**Figure 2:**
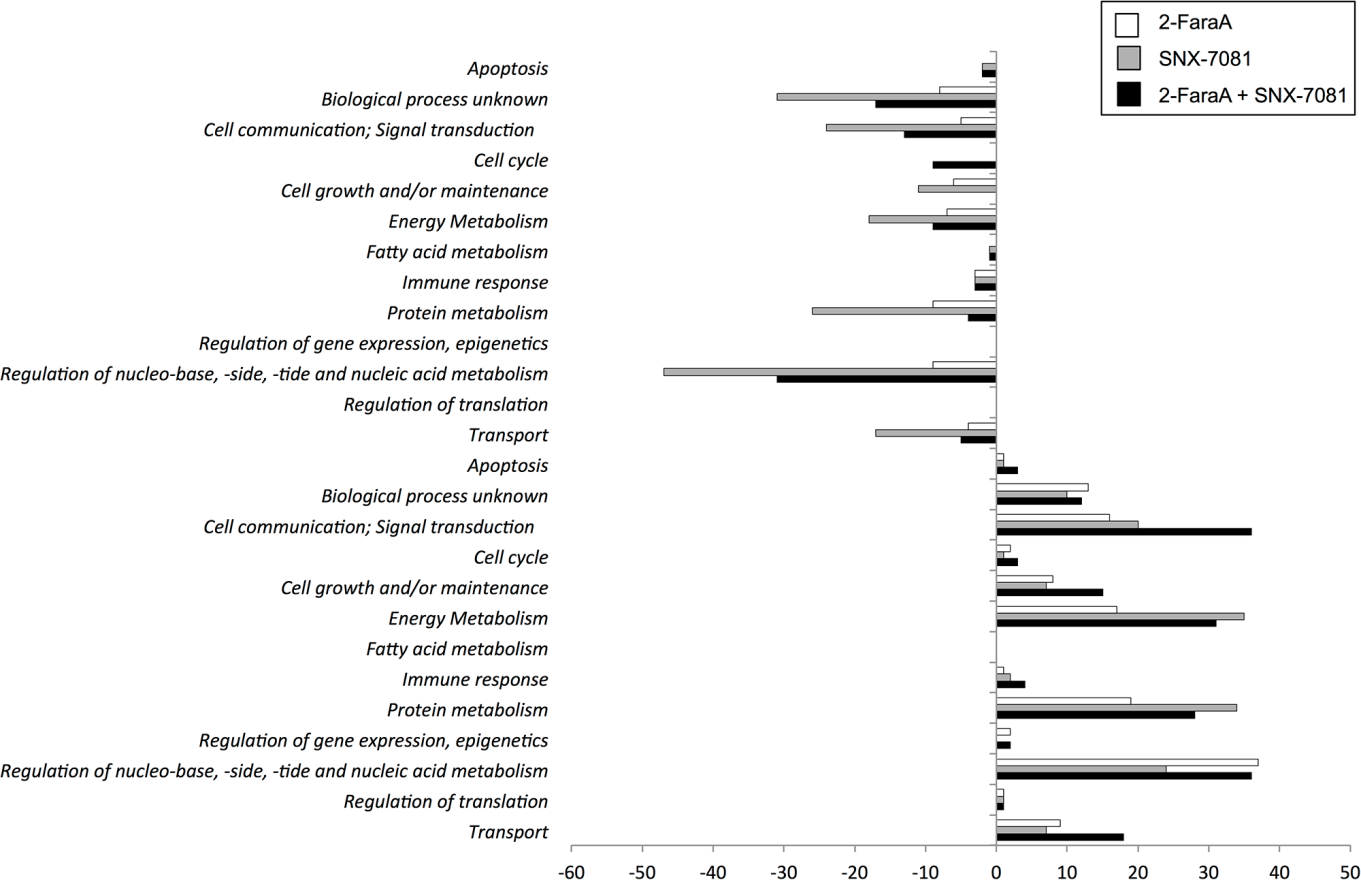
Comparison and classification of MEC1 cell proteins that changed more than 2-ratio (*p* < 0.05) after 2-FaraA (10 μM, 48 h), SNX-7081 (100 nM, 48 h), and SNX-7081 (100 nM) + 2-FaraA (10 μM) (48 h) The Human Protein Reference Database classified proteins into 13 different biological process categories, the numbers of proteins are indicated as bars. The bars with negative values on the x-axis indicate the number of proteins decreasing after drug treatment. Conversely the bars with positive values indicate numbers of proteins that increased in abundance.

Protein abundances were compared between control and drug-treated cells to identify protein changes following single or dual treatments. Differentially abundant proteins that changed by more than 2-ratio (*p* < 0.05) are summarized in Figure 1 and Table 2. Dual treatment increased the abundance of 189 proteins, 83 and 67 of which also increased after SNX-7081 and 2-FaraA treatments alone. Levels of 94 proteins decreased following dual drug treatment, 73 and 21 of which decreased after SNX-7081 or 2-FaraA, respectively. Overall, 62 proteins changed (43 increased and 19 decreased) across all three datasets. A master list of identified protein changes is provided in Supplementary Table 1. Proteins were grouped based on their predominant biological process, according to the Human Protein Reference Database (http://www.hprd.org/; Figure 2).

**Table 2:** Number of differntially abundant proteins induced by single or dual drug treatment ratio of-change > 2, *p* < 0.05)

Comparative analysis	Abundance increased	Abundance decreased	Total changes
Control vs. 10 μM 2-FaraA-treated (48 h)	126	51	177
Control vs. 100 nM SNX-7081-treated (48 h)	140	182	322
Control vs. 10 μM 2-FaraA + 100 nM SNX-7081 (48 h) treated	189	94	283

### Proteome changes induced in MEC1 cells by 2-FaraA

**Figure 3:**
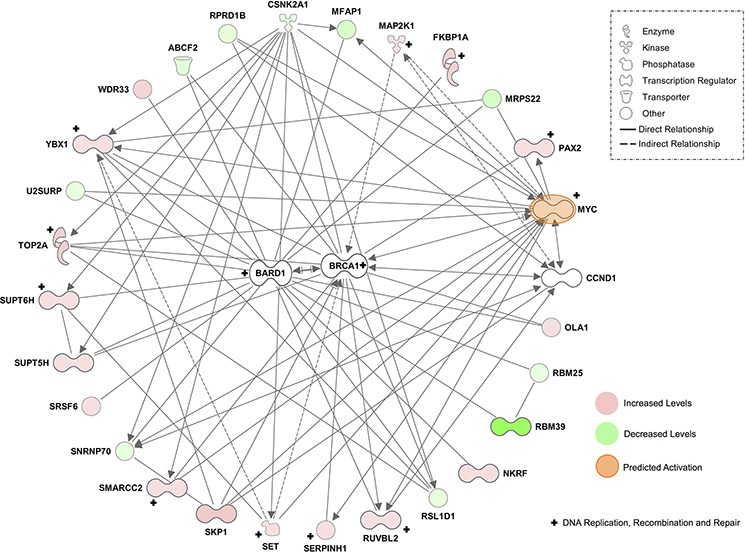
Simplified radial network based on the top scoring protein interaction network of proteins changing after 2-FaraA treatment (*51 focus molecules*) This network was generated in the IPA environment and converges on the heterodimer BRCA1/BARD1, with prominent MYC connectivity. Quantitative proteomic analyses identified proteins increased by > 2-ratio (red) and decreased by < 0.5-ratio (green, *p* < 0.05). IPA predicted the activation (orange) of MYC in this network. Molecules functionally annotated by DNA replication, recombination and repair are indicated by ‘+’.

2-FaraA induced the fewest protein changes in MEC1 cells, consistent with previous reports of resistance to 2-FaraA [18]. Overall, 177 proteins changed, of which 126 increased and 51 decreased after 2-FaraA (10 μM, 48 h; *p* < 0.05) compared with untreated controls. Proteins with increased abundance following 2-FaraA treatment were predominantly involved in nucleobase, nucleoside, nucleotide and nucleic acid metabolism (37 proteins), including the DNA damage protein TOP2A (12.5-ratio) and proteins positively regulating DNA replication and repair (SSBP1, 21.0-ratio; SET, 2.46-ratio; POLD, 2.27-ratio; RUVBL2, 2.03-ratio). Proteins related to cell cycle progression were also increased after 2-FaraA (SKP1, 14.30-ratio; ANAPC5, 9.87-ratio; PARP10, 7.65-ratio; SEPTIN11, 2.47). A simplified radial interaction network converging on the heterodimer BRCA1 and BARD1, with prominent MYC connectivity (predicted upstream activation, z-score = 2.202, *p*-value = 1.95E^−04^), is provided in Figure 3.

### Proteome changes induced in MEC1 cells by SNX-7081

We identified 322 protein changes (140 increased and 182 decreased, Supplementary Table 1) in SNX-7081-treated MEC1 cells compared with untreated cells (*p* < 0.05). Proteins with increased levels following SNX-7081 included those involved in energy (35) and protein (34) metabolism. Proteins with reduced levels following SNX-7081 were predominantly involved in nucleoside, nucleotide and nucleic acid metabolism (48 molecules), including several decreased proteins that positively regulate DNA replication and repair (MCM6, 0.17-ratio; MCM7, 0.20-ratio; MCM2, 0.41-ratio; MCM5, 0.42-ratio; SSRP1, 0.15-ratio; RRM2, 0.29-ratio; NONO, 0.50-ratio; XRCC5, 0.50-ratio; FEN1, 0.50-ratio; FUS, 0.49-ratio).

### Proteome changes induced in MEC1 cells by dual drug treatment

Quantitative proteomic analysis of MEC1 cells following dual drug treatment (100 nM SNX-7081 + 10 μM 2-FaraA, 48 h) identified 282 differentially abundant proteins (189 increased, and 94 decreased by more than 2-ratio, *p* < 0.05) compared with untreated controls. Proteins identified at higher levels (36) were predominantly involved in nucleobase, nucleoside, nucleotide and nucleic acid metabolism, including increased levels of DNA damage proteins TOP2A (6.5-ratio) and TOP2B (6.0-ratio). Levels of 31 proteins in this functional group decreased after dual treatment, including several proteins that positively regulate DNA replication and repair (MSH6, 0.22-ratio; RFC5, 0.06-ratio; MCM6, 0.30-ratio; RFC4, 0.15-ratio; MCM7, 0.40-ratio; TP53BP1, 0.50-ratio; XRCC5, 0.50-ratio). Proteins related to gene expression/epigenetic regulation (PML, 65.45-ratio; histone H2A.V, 52.70-ratio), cell cycle (SKP1, 11.08-ratio; SEPT11, 2.35-ratio; YWHAZ, 2.43-ratio) and apoptosis (BID, 18.72-ratio; MZB1, 7.68-ratio; FAF2, 3.07-ratio) were also increased following dual treatment.

### Interaction networks predicted changes in activation states of up-stream regulators

Genes corresponding to all differentially abundant proteins were mapped in the IPA environment; summaries of biological and molecular associations are provided in Table 3. An interaction network comprising 58 molecules was generated, including 12 proteins that were significantly affected by 2-FaraA treatment, 20 by SNX-7081 and 35 that changed following dual treatment. Networks illustrating the protein levels measured by quantitative MS and predicted activation states across the three treatment conditions are provided in Supplementary Figure 1A, 1B and 1C. Molecules previously linked to ‘B-Cell lymphoproliferative disorders’, ‘MYC-mediated apoptosis signaling’, ‘DNA damage’ and ‘DNA damage checkpoint regulation’ are also annotated. Hsp90 inhibition was predicted after overlaying the SNX-7081 and dual treatment proteomic data onto this interaction network. Likewise, the DNA damage marker H2AX was activated when the dual treatment dataset was overlaid. MYC, a predicted upstream regulator in all three datasets, was predicted to be active after 2-FaraA treatment and inhibited following SNX-7081 and dual treatment. CCND1 was predicted as inhibited in the 2-FaraA and dual treatments but activated after SNX-7081 alone. Inhibition of FAS death receptor and caspase 8 after 2-FaraA, and activations after SNX-7081 and dual drug treatment were also projected by the network.

**Table 3:** Biological and molecular functions significantly associated with the proteomics datasets

*Molecular and Cellular Functions*		*Molecules*	*p-value*
2-FaraA	RNA post-transcriptional modification (*processing of RNA)*	19	8.97E^−11^
	Gene expression (*expression of RNA)*	43	4.54E^−05^
SNX-7081	RNA post-transcriptional modification (*processing of RNA)*	27	3.56E^−12^
	Protein synthesis (*metabolism of protein)*	45	1.39E^−09^
	Gene expression (*expression of mRNA)*	18	2.38E^−08^
2-FaraA+ SNX-7081	RNA post-transcriptional modification (*processing of RNA)*	23	2.70E^−10^
	Cellular growth and proliferation (*proliferation of cells)*	87	2.94E^−07^
***Top Scoring Networks***		***Molecules***	***Score***
2-FaraA	Cancer, cell death and survival	51	66
	Cellular development, cellular growth and proliferation, hematological system development and function	46	57
SNX-7081	DNA replication, recombination and repair, cellular growth and proliferation, cellular development	83	107
	DNA replication, recombination and repair, cell death and survival, cell cycle	61	58
2-FaraA+ SNX-7081	Cellular growth and proliferation, cell death and survival, cellular development	71	87
	Cell death and survival, organismal survival, cellular development	57	62
***Top Canonical Pathways***		***Overlap***	***p-value***
2-FaraA	Protein ubiquitination pathway	14/242	5.24E^−08^
	tRNA charging	5/39	3.57E^−05^
	EIF2 Signalling	9/183	2.75E^−04^
	Regulation of eIF4 and p70S6K signalling	7/143	2.75E^−04^
SNX-7081	Regulation of eIF4 and p70S6K signalling	19/143	1.58E^−12^
	EIF2 Signalling	21/183	1.91E^−09^
	mTOR signalling	18/185	1.15E^−09^
	tRNA charging	8/39	1.59E^−07^
2-FaraA+ SNX-7081	EIF2 signalling	14/183	3.33E^−07^
	Regulation of eIF4 and p70S6K signalling	11/143	5.84E^−06^
	G2/M DNA checkpoint regulation	6/49	6.16E^−05^
***Upstream Regulators***		***Molecules***	***p-value***
2-FaraA	MYC *Predicted activation (z-score = 2.202)*	12	1.95E^−04^
	HSF1	5	1.75E^−03^
	XBP1	5	6.31E^−03^
SNX-7081	E2F1	16	4.90E^−07^
	E2F4	13	5.03E^−07^
	HSF1	8	1.81E^−04^
	XBP1	9	2.85E^−04^
	MYC	15	1.77E^−03^
2-FaraA+ SNX-7081	MYC	16	1.47E^−04^
	E2F1	12	6.16E^−05^
	HSF1	9	9.94E^−06^
	MAPT	5	6.30E^−05^

### Confirmations of identified and predicted proteins changes

**Figure 4:**
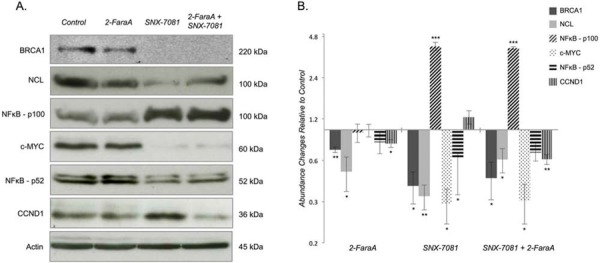
Western blot analysis of identified and predicted protein changes in MEC1 cells treated with 2-FaraA, SNX-7081 or 2-FaraA + SNX-7081 **A.** Western blots of BRCA1, NCL, NFkB p100/p52, MYC and CCND1 in MEC1 untreated and treated cells. **B.** Abundance changes relative to control cultures are graphed on a log2 scale. Relative band intensities were quantified using ImageJ software and biological triplicates were normalized to β-actin internal control and averaged. Error bars represent mean ± standard deviation; significant changes are indicated by *p < 0.05, **p < 0.01, ***p < 0.0001.

Increases in NFκB2 p100 levels following SNX-7018 (4.06-ratio, *p* = 5.84E^−05^) and dual treatment (3.94-ratio, *p* = 9.74E^−07^) were confirmed by Western blot. While concomitant decreases in NFκB2 p52 levels were detected following all treatment conditions, only SNX-7081 induced a significant change (0.62-ratio, *p* = 0.022; Figure 4). We also confirmed decreases in NCL following SNX-7018 and dual treatments (0.33-ratio, *p* = 0.004 and 0.61-ratio, *p* = 0.014, respectively); reduced NCL levels were also detected following 2-FaraA (0.50-ratio, *p* = 0.047; Figure 4). All IPA core analyses independently implicated MYC (myelocytomatosis oncogene cellular homolog, transcription factor p62) as an upstream regulator of proteins changing in response to each treatment condition. Western blot analysis showed that 2-FaraA had no effect on MYC levels (0.98-ratio); SNX-7081 alone and in combination with 2-FaraA induced significant decreases in MYC (0.29-ratio, 0.30-ratio, *p* = 0.013, *p* = 0.011, respectively; Figure 4). IPA also revealed interactions with cyclin D1 (CCND1) and predicted its inhibition following 2-FaraA and dual treatment, and activation following SNX-7081, in line with our previous study [16]. Western blot analysis showed decreases in CCND1 following 2-FaraA and dual treatment (0.90-ratio, *p* = 0.039 and 0.49-ratio, *p* = 0.007, respectively; Figure 4). A non-significant increase in CCND1 was detected after SNX-7081 (1.24-ratio, *p* = 0.187). BRCA1 was also detected at reduced levels following all three treatments, relative to control cultures (0.72-ratio, *p* = 0.004; 0.39-ratio, *p* = 0.023; 0.45-ratio, *p* = 0.047 following 2-FaraA, SNX-7081 and dual treatment respectively; Figure 4).

### Synergistic treatment increases DNA damage in p53-mutated *human B-lymphoid cancer cells*

**Figure 5:**
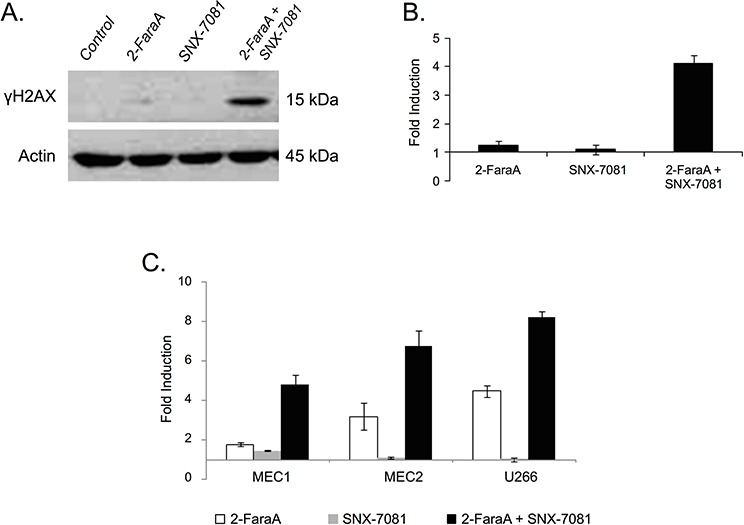
Levels of the DNA damage marker γH2AX in response to drug treatments **A, B.** Western blot analysis of γH2AX levels induced by 2-FaraA (10 μM, 48 h), SNX-7081 (100 nM, 48 h), and 2-FaraA (10 μM) + SNX-7081 (100 nM) (48 h) compared with control cells. **C.** Flow cytometry analysis of γH2AX levels compared with controls for the cell lines, MEC1, MEC2 and U266 after 2-FaraA (10 μM, 48 h), SNX-7081 (100 nM, 48 h), and FdA (10 μM) + SNX-7081 (100 nM) (48 h). The level of actin was used to normalize protein levels for control and treated cells. Fold-induction was calculated as the intensity of the treated sample divided by the intensity of the control. Error bars show mean ± standard deviation. The values plotted are means from 3 independent experiments.

Levels of the phosphorylated form of the histone H2AX (γH2AX), a marker of DNA DSBs [19], were measured following 2-FaraA, SNX-7081, and dual drug treatment. Following 2-FaraA treatment the levels of γH2AX in MEC1 cells increased by 1.3-ratio, SNX-7081 induced a 1.1-ratio increase, while combination of the two drugs resulted in a synergistic increase of 4.1-ratio (Figure 5A and 5B). Flow cytometry confirmed these results; γH2AX increased by 1.8-ratio following 2-FaraA, 1.5-ratio following SNX-7081, and 4.8-ratio after dual treatment (Figure 5C). Similar synergy was seen for the additional p53-mutated B-lymphoid cell lines, MEC2 and U266. In MEC2 cells, changes in γH2AX were 3.21-ratio, 1.10-ratio and 6.75-ratio following 2-FaraA, SNX-7081 and dual treatment, respectively. In U266 cells, the respective treatments induced γH2AX by 4.5-ratio, 1.0-ratio and 8.3-ratio.

## DISCUSSION

We previously reported that the HSP90 inhibitor, SNX-7081 synergizes with and restores sensitivity to 2-FaraA, by inducing apoptosis in refractory CLL cells [10], and proposed that this was facilitated by SNX-7081-induced inhibition of the cell cycle, DNA replication and repair [16]. To substantiate these findings, we have expanded our proteomics analysis to quantitate protein changes in MEC1 cells following 2-FaraA + SNX-7081 treatment to describe the mechanism underpinning this dual therapy.

### Enhanced DNA replication and repair underpins MEC1 resistance to 2-FaraA

Cells with mutations in p53 (e.g., MEC1) are generally resistant to DNA damaging agents such as 2-FaraA [7, 8], due to their inability to induce apoptosis and down-regulate DNA repair proteins [9]. In the absence of functional p53, 2-FaraA increased levels of the DNA damage and repair proteins (TOP2A, POLD1, SSBP1, SET and RUVBL2) and cell cycle proteins (SKP1, ANAPC5, PARP10 and SEPTIN11) in MEC1 cells. These increases likely enable MEC1 cells to repair 2-FaraA-induced DNA damage and resist apoptosis [20], consistent with the promotion of DNA replication and repair contributing to 2-FaraA resistance.

IPA predicted that MYC was active in 2-FaraA-treated MEC1 cells owing to significant changes in 12 downstream proteins: increases in AK2, CSDE1, GLUD1, MAT2A, PAX2, POLD1, SLC16A1, SLC38A1, SLC7A5 and decreases in C1QBP, GAPDH and MFAP1. Increased MYC was observed in clinical samples of B-CLL cells resistant to 2-FaraA; a MYC-specific regulatory network was proposed as the mechanism of resistance to 2-FaraA [21]. Interestingly, Jitschin *et al.*, recently showed that Notch-mediated MYC expression in CLL cells is important in the tumour microenvironment, contributing to the metabolic alterations seen in treatment resistance [22]. The down regulation of MYC may therefore present a therapeutic strategy in targeting the proliferative compartment of this CLL. The role of MYC in apoptosis signalling following DNA damage is poorly understood. Cross-talk between MYC and p53 is important for regulating cell fate decisions, but MYC-mediated apoptosis can also be p53-independent. In lymphoid and myeloid cells, MYC-driven apoptosis appears to be p53-independent [23, 24]. While 2-FaraA did not increase MYC levels in our experiments, the sustained MYC levels observed (Figure 4) could mediate apoptosis resistance in p53-negative MEC1 CLL cells.

The tumor suppressor BRCA1, along with heterodimer partner BARD1 (BRCA1-associated RING domain protein), functions in a variety of cellular processes including DNA repair, cell cycle checkpoint activation, apoptosis and the transcriptional regulation of genes associated with these pathways [25, 26]. BRCA1/BARD1 localizes to the centrosome throughout the cell cycle [27] and appears to function upstream of DNA repair pathways, including the two major repair pathways, non-homologous end joining (NHEJ) and homologous recombination (HR) [28], possibly acting as a sensor for DNA damage. Multiple studies have demonstrated that BRCA1 mutantor deficient cells are hypersensitive to topoisomerase inhibitors and cross-linking agents [29–31] that damage DNA. Resistance to DNA damage mediated by BRCA1 correlates with a suppression of apoptosis [30, 32]. BRCA1/BARD1 was not detected by LC-MS/MS, however IPA revealed prominent connections with 26 proteins that significantly changed following 2-FaraA, 15 of which also interact with MYC (Figure 4), suggested that BRCA1 is an important mediator of 2-FaraA resistance in p53-negative CLL. However, 2-FaraA induced a significant reduction in BRCA1 (0.72-ratio), albeit less dramatically than SNX-7081 and dual treatment (0.39-ratio and 0.45-ratio decreases, respectively), and reductions in BRCA1 would render MEC1 cells more susceptible to DNA damage-related death. The 1.3-ratio increase in γH2AX levels in 2-FaraA-treated MEC1 cells suggests that some DNA damage occurs but is limited by enhanced DNA repair. BRCA1 is therefore unlikely to be a principal mediator of 2-FaraA resistance in MEC1 cells.

IPA also predicted an association between 2-FaraA-induced changes and the protein ubiquitination pathway (increases in ANAPC5, B2M, DNAJB11, PSMA4, PSMB1, PSMD12, SKP1, TCEB1, UBE2V1, USP15, USP47, decreases in PSMC6, PSMD7 and USP39). There is mounting evidence that ubiquitin family members are also strong regulators of DNA repair in chemo-resistant cancer cells [33], through mechanisms such as nucleotide excision repair, post-replication repair and HR [34]. tRNA charging, that is the loading of specific cognate amino acids to amino acyl tRNA synthetases (AARS), was also significantly associated with 2-FaraA protein changes. Mammalian AARSs have evolved additional domains that enable them to interact with various proteins, some of which are implicated in tumorigenesis. We previously identified changes in six AARS proteins in PI3K-inhibited colorectal cancer cells, not related to their traditional roles in protein synthesis [35]. Interestingly, Wei *et al.*, demonstrated a novel role for tyrosyl-tRNA synthetase (YARS) in DNA damage protection [36]. The detected increase in YARS2 (8.85-ratio) may assist MEC1 cells in resisting 2-FaraA-induced DNA damage.

### HSP90 inhibition by SNX-7081 decreases DNA repair proteins alone and in combination with 2-FaraA

Hsp90 is a molecular chaperone required for the folding and function of multiple signaling proteins that promote growth and survival in cancer cells. We previously reported that Hsp90-inhibition in MEC1 cells reduces the levels of cell cycle proteins, proteins involved in DNA replication and repair, RNA processing and regulation of transcription and translation [16]. Using an alternative quantitative mass spectroscopy approach, we have detected increases in chaperones (HSPH1, HSPB1, DNAJB1) as well as decreases in nuclear pore proteins (NUP50, NUP93, NUP155), DNA replication licensing factors (MCM2, MCM6, MCM7), RNA helicase protein, SNRNP200 and ribosome biogenesis factor, NOL11, following SNX-7081 treatment. The signal transduction protein, PRKDC, showed a major decrease (0.0030-ratio) after SNX-7081. PRKDC is a DNA-dependent serine/threonine-protein kinase (DNA-PK) that acts as a molecular sensor of DNA damage [37] and assembly of the DNA-PK complex at DNA strand breaks is required for the NHEJ ligation step [38]. X-ray repair cross-complementing protein 5 (XRCC5) was also significantly reduced (0.5-ratio) following both SNX-7081 and dual treatment. The XRCC5/6 dimer stabilizes broken DNA ends, an important step in DNA NHEJ required for DSBs and V(D)J recombination [39–41], and acts as a regulatory subunit of the DNA-PK, increasing the affinity of PRKDC to DNA. The disappearance of PRKDC and XRCC5 strongly suggests that SNX-7081 inhibits the response to DNA damage.

Several proteins that positively regulate DNA replication and repair were significantly decreased following dual drug treatment (MSH6, RFC4, RFC5, MCM6, MCM7, TP53BP1). MSH6 is a component of the post-replicative DNA mismatch repair system (MMR) that hetero-dimerizes with MSH2 to form MutS alpha. This dimer then binds to DNA mismatches and initiates DNA repair. MSH6 also interacts with proliferating cell nuclear antigen [42–44] (PCNA; nuclear levels significantly reduced by SNX-7081 [16]) and is part of the BASC (BRCA1-associated genome surveillance complex) involved in DNA repair [45]. RFC4 and RFC5 are two of the five-subunit RFC (replication factor C) complex used in eukaryotic replication as a clamp loader for loading PCNA onto DNA [46], required for DNA replication. Several components of the MCM complex that forms the replicative helicase essential for ‘once per cell cycle’ DNA replication initiation and elongation in eukaryotic cells [47], decreased after SNX-7081 and in combination with 2-FaraA.

Promyelocytic leukemia protein (PML) is a key component of PML nuclear bodies; dynamic nuclear aggregates are suggested to be sites of epigenetic regulation and sensors of DNA damage [48]. PML influences MYC transcriptional activity through a mechanism that involves control of post-translational modifications of MYC [49]. The major increase in PML following SNX-7081+2-FaraA (65.4-ratio) indicates major DNA damage. Histone H2A family proteins are important for packaging DNA into chromatin, and have been associated with DNA modification and epigenetics. The enzymes, TOP2A and TOP2B, are involved in formation DNA DSBs [50]. The increases of TOP2A and TOP2B after the dual treatment (6.51-ratio and 5.98-ratio, respectively) suggest that in addition to incorporation of 2-FaraATP into elongating DNA chains, 2-FaraA may increase proteins involved in formation of the DNA strand-breaks.

**Figure 6:**
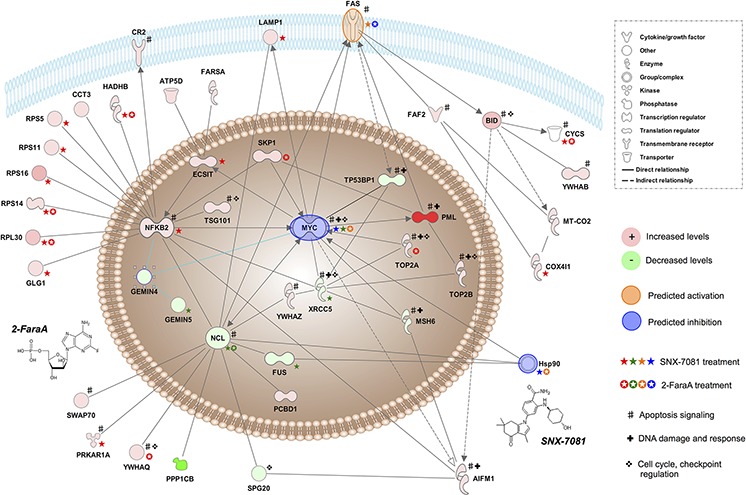
Simplified network of key interactions underpinning 2-FaraA + SNX-7081 synergy This network describes the proposed mechanism of MEC1 cell death and was generated by expanding interactions between molecules, NFκB2, MYC and NCL and other significant protein abundance changes following synergistic drug treatment. Proteins with increased levels (>2-ratio) after drug treatment are in red and proteins with decreased levels (<0.5-ratio) are in green, more pronounced changes are indicated by more saturated colors. Predicted activations are in orange, and predicted inhibitions are in blue. Molecules previously linked to apoptosis signaling, DNA damage and response and cell cycle, DNA damage checkpoint regulation are annotated. Changes in protein abundance (detected by mass spectrometry and Western blot) induced by SNX-7081 are indicated by a star, and induced by 2-FaraA by an enclosed star; increases, decreases, predicted activation and inhibition are color-coded as above. Decreased MYC levels (Figure 4) supports the predicted inhibition of MYC here.

SNX-7081 and 2-FaraA synergy is further supported by the observed changes in γH2AX levels. Phosphorylation of H2AX on serine 139 is a marker of DNA DSBs [51]. We demonstrated a significant increase in γH2AX in MEC1 cells treated with SNX-7081+2-FaraA, compared with exposure to either agent alone (Figure 5A). These data suggest that some DNA damage does occur following exposure to 2-FaraA (1.3-ratio increase), however cells resist apoptosis by enhancing DNA repair machinery. The high levels of γH2AX in dual treated cells supports the hypothesis that SNX-7081 hinders the repair of DNA breaks induced by 2-FaraA, thus prolonging H2AX phosphorylation. The additional p53-mutated cell lines, MEC2 and U266, showed similar changes in γH2AX (Figure 5C), suggesting that this is a general drug synergy mechanism in p53-mutated B-lymphoid cancer cell lines [10]. The H2AX molecule was added to the network in Figure 6; the predicted activation following synergistic drug treatment supports the findings of increased levels of phosphorylation.

There are some differences between significant proteins identified here, and those previously reported [16]. This includes, but is not restricted to, the non-significant increase of CCND1 following SNX-7081 here. This, and others may be explained by several factors including the whole cell proteome analysis approach employed here, as opposed to the sub-cellular proteome analyses conducted before. Whole cell analyses do not resolve changes in protein levels due to shuttling between subcellular compartments. Perhaps of more consequence, MEC1 cells were treated with a low SNX-7081 dose here (100 nM), compared to treatment at the IC_50_ (500 nM) as before.

### Hsp90 inhibition suppresses MYC and causes NFkB2 p100 accumulation

The transcription factor MYC is involved in cell proliferation and apoptosis and plays a direct role in the control of DNA replication. The decrease of MYC might contribute to decreased DNA repair and replication capacity of Hsp90-inhibited cells. Significant changes in 15 proteins had predicted upstream regulation by MYC; Western blot analysis confirmed that MYC is reduced following HSP90 inhibition alone or in combination with 2-FaraA (see Figure 4). Reduced MYC was also reported following Hsp90 inhibition of neuroblastoma and prostate cancer cells, with suppression of the malignant phenotype and augmentation of chemotherapy response, respectively [52, 53]. We previously showed that SNX-7081 modulates a number of proteins at the mRNA level (PCNA, MCM2, Nup155, Hsp70, GRP78, PDIA6, and HLA-DR) [16], that might be caused by a deregulation of gene expression and mRNA methylation as a consequence of MYC destabilization [54].

Nuclear factor-kappa-B p100 subunit (NF*k*B2) was identified as a MYC repression target; MYC appears to regulate both basal and stimulated NF*k*B2 transcription. In Eμ-MYC transgenic mice, where MYC overexpression drives B-cell lymphoma, NFκB2 loss accelerates tumor development by impairing MYC-dependent apoptosis, highlighting the tumor suppressor function of the non-canonical NFκB pathway [55]. The NFκB signaling cascade can be divided into two pathways depending on how active homo-/heterodimers are produced. In the non-canonical (alternative) NFκB pathway, activation is achieved by inducing post-translational processing of p100 to the p52 subunit. Here, p100 is phosphorylated, leading to poly-ubiquitination and proteasomal processing to p52, which forms the transcriptionally active p52/RelB dimer [56]. NFκB2 appears to have dual functions, including cytoplasmic retention of attached NF-*k*B proteins by p100 and generation of p52 by a co-translational processing. Inactive NF*k*B dimers are sequestered in the cytoplasm due to their interaction with inhibitory proteins, including NF*k*B p100. In lymphoma cells, the accumulation of p105 and p100 is essential for the induction of apoptosis produced by proteasome inhibition [57]. The Hsp90 inhibitor, NVP-AUY922-AG decreased transcription of NF*k*B target genes, and this effect was maintained for synergistic dual treatment with 2-FaraA of primary CLL cells [58]. We detected significant increases in NF*k*B2 p100 after SNX-7081 and dual treatment, confirmed by Western blot (Figure 4). Interestingly, we also detected a significant, concomitant decrease in NF*k*B2 p52 subunit following SNX-7081 (Figure 4), implicating an HSP90-related inhibition of p100 proteasome processing and accumulation of the apoptosis-inducing p100 subunit.

NF*k*B2 interacts with nucleolin (NCL), a highly conserved, ubiquitous, anti-apoptotic protein that is expressed by exponentially growing eukaryotic cells [59]. NCL is also involved in regulating multiple apoptosis-related molecules [60] and is a novel modulator of the Fas death receptor in human lymphomas, effectively blocking Fas signaling [61]. Hsp90 was previously shown to stabilize NCL [62] and further, NCL knockdown can inhibit DNA-PK phosphorylation, thereby reducing DNA DSB repair [59]. Here, Hsp90 inhibition alone and in combination with 2-FaraA significantly reduced NCL levels, measured by mass spectrometry and Western blot (see Figure 4), potentially sensitizing MEC1 cells to Fas-induced apoptosis. NCL levels also decreased following 2-FaraA as measured by Western blot (Figure 4), implicating other intermediary and/or regulatory molecules in MEC1 resistance to 2-FaraA.

### Simplified mechanism for synergic killing of MEC1 cells

A simplified interaction network, presented in Figure 6, details the proposed mechanism of 2-FaraA and SNX-7081 synergistic killing of p53-negative MEC1 cells. Accumulating DNA damage, indicated by the synergistic increase in γH2AX levels, is accentuated by SNX-7081-mediated inhibition of DNA repair, resulting in the enhanced induction of apoptosis previously reported [10]. Our data suggest that DNA damage is compounded by the loss of multiple DNA damage response molecules, including checkpoint regulators BRCA1 and CCND1, and cell death is triggered following a loss of MYC and NCL and an accumulation of NF*k*B2 p100. Loss of NCL can activate Fas-mediated apoptosis, leading to increases in pro-apoptotic proteins BID and fas-associated factor 2 (FAF2). While observed changes in BRCA1, CCND1, MYC, NCL, NF*k*B2 p100, BID and FAF2 correlate well with the known functions of these proteins, further delineation of the causes and effects of each modulated protein is required.

## MATERIALS AND METHODS

### Cell culture and drug treatment

The CLL cell lines, MEC1 and MEC2, have a deletion in the short arm of chromosome 17 (17*p*-), the locus for p53, with an N-terminal truncation mutation in the other allele. These cell lines are therefore suitable models for testing drugs for the treatment of p53 mutant CLL [63]. The human U266 myeloma cell line has a mutation in p53 at codon 161 [64]. Human MEC1, MEC2, and U266 cells were grown in RPMI 1640 medium supplemented with 10% fetal calf serum and 50 μg/mL gentamicin at 37°C. Cells were grown in exponential phase and treated with drugs at a density of 3 × 10^5^ cells/ml. SNX-7081 (a kind gift from Prof. Lee Graves, Department of Pharmacology, University of North Carolina, Chapel Hill, NC, USA) and/or 2-FaraA (Sigma-Aldrich, St. Louis, MO, USA) were dissolved in DMSO. From previous experiments, combining 2-FaraA (10 μM, 48 h) and SNX-7081 (100 nM, 48 h) gave the greatest effect against B-lymphoid MEC1, MEC2 and U266 cells [10]. Triplicate cultures of MEC1 cells were treated: control (DMSO, 48 h), 2-FaraA (10 μM, 48 h), SNX-7081 (100 nM, 48 h), 100 nM SNX-7081 + 10 μM 2-FaraA (48 h). Control cultures were incubated with the same amount of DMSO as a vehicle control.

### Protein extraction, SDS-PAGE separation and in-gel tryptic digestion

MEC1 cells were collected from triplicate cultures (see above) by centrifugation (350 x *g*, 5 min, 4°C) and lysed in protein extraction buffer (5 M urea, 2 M thiourea, 2% w/v CHAPS, 2% w/v sulfobetaine 3–10, 40 mM Tris-HCl (pH 8.8), 1.0% v/v carrier ampholyte, 65 mM DTT, 2 mM tributylphosphine). The lysate was centrifuged (10,000 × *g*, 5 min, 4°C) and the supernatant purified by precipitation (Ready Prep 2-D Clean-up Kit, Bio-Rad Laboratories, Hercules, CA, USA) and re-suspended in 8 M urea. Protein concentrations were determined (2D Quant Kit, GE Healthcare, Little Chalfont, Buckinghamshire, UK) and 200 μg samples were diluted in sample buffer (40% glycerol, 240 mM Tris-HCl pH 6.8, 8% SDS, 0.04% bromophenol blue, 5% β-mercaptoethanol) (200 μg protein per well) and separated by 10% PAGE at 100 V for 1.5 h. Coomassie blue-stained SDS-PAGE gels were washed and each lane was cut into 16 equal pieces, each piece then sliced finely and transferred to a 96-well plate. Gel fragments were de-stained with 100 mM NH_4_HCO_3_, and dehydrated with acetonitrile (ACN, 50% v/v) in 100 mM NH_4_HCO_3_ for 10 min, then 100% ACN for 10 min before air drying. The samples were reduced with 10 mM DTT at 37°C for 1 h, followed by alkylation in the dark with 50 mM iodoacetamide at room temperature for 1 h. The samples were then washed and dehydrated and air-dried as before. Samples were rehydrated with sequencing grade trypsin (Promega, Madison, WI, USA) at a trypsin to protein ratio of 1:10 for 30 min on ice and then allowed to digest overnight (37°C). Peptide mixtures were extracted twice with ACN (50% v/v) in 2% formic acid, dried by vacuum centrifugation and reconstituted with 2% formic acid in H_2_O.

### Nano liquid chromatography-tandem mass spectrometry

As previously described [65], peptides were analyzed by nanoLC-MS/MS using a LTQ-XL ion-trap mass spectrometer (Thermo Fisher Scientific, San Jose, CA, USA). Reverse phase columns were packed in-house to approximately 7 cm (100 μm i.d.) using 100 Å, 5 μm Zorbax C18 resin (Agilent Technologies, Santa Clara, CA, USA) in a fused silica capillary with an integrated electrospray tip. A 1.8 kV electrospray voltage was applied via a liquid junction up-stream of the C18 column. Samples were injected onto the C18 column using a Surveyor autosampler (Thermo Fisher Scientific), followed by an initial wash with buffer A (5% (v/v) ACN, 0.1% (v/v) formic acid) for 10 min at 1 μL/min. Peptides were eluted from the C18 column with 0%-50% Buffer B (95% (v/v) ACN, 0.1% (v/v) formic acid) over 58 min at 500 nL/min followed by 50–95% Buffer B over 5 min at 500 nL/min. The eluted solution was directed into the nanospray ionization source of the mass spectrometer. Spectra were scanned over the range 400–1500 amu, with automated peak recognition, a dynamic exclusion window set to 90 s and tandem MS of the top 6 most intense precursor ions at 35% normalization collision energy using Xcalibur software (version 2.06; Thermo Fisher Scientific).

### Protein identification and data processing

MS/MS raw data files were converted to mzXML format and processed through the global proteome machine (GPM) software using version 2.1.1 of the X!Tandem algorithm (http://www.thegpm.org). For each experiment, the 16 fractions were processed sequentially with output files for each fraction, and a merged, non-redundant output file was generated for protein identifications with log(e) values less than −1. Tandem mass spectra were searched against the NCBI human protein database in the Global Proteome Machine containing 513,692 protein sequences. The database also incorporated common human and trypsin peptide contaminants. Search parameters included MS and MS/MS tolerances of ± 2 Da and ± 0.2 Da, respectively and up to three missed tryptic cleavages and K/R-P cleavages. Fixed modifications were set for carbamidomethylation of cysteine and variable modifications were set for oxidation of methionine. Triplicate GPM protein identification files were combined into one joint dataset to produce a single shotgun proteomic analysis for each condition. Only proteins identified in all replicates were retained in the final dataset for each condition. To enhance confidence, a total spectral count of at least six was required within at least one condition.

### Quantitative proteomics and statistical analyses

Protein abundance data were calculated based on normalized spectral abundance factor (NSAF) values [66] and following the workflow and software previously described [67]. For each sample, *i*, the number of spectral counts (*SpC*) identifying a protein, *k*, was divided by the molecular weight of the protein in kDa. (SpC/MW)*k* values were then divided by the sum of (SpC/MW) for all (*N*) proteins in the experiments to give the NSAF*i* values. For each protein *k*, the sum_*S*_ of all spectral counts obtained from the triplicate values was calculated, and the corresponding NSAF*_S_*values were deduced and used as a measure of protein abundance. A spectral fraction of 0.5 was added to the spectral counts of each protein, to compensate for null values [68] and enable log-transformation for subsequent statistical analyses. Two-tailed *t*-tests assuming equal variance were performed on log_2_-transformed NSAF data to identify significant protein changes following each treatment, where *p* < 0.05 was regarded as significant. Proteins that had a ratio of change greater than 2, or less than 0.5 were analyzed further.

### Bioinformatics

Biological and canonical functions of differentially abundant proteins across the three datasets were explored using Ingenuity® Pathway Analysis (IPA) software (Ingenuity Systems, http://analysis.ingenuity.com). This software program calculates the probability that the genes associated with our datasets (right-tailed Fisher's Exact Test) are involved in particular pathways, compared with the total number of occurrences of those proteins in all functional annotations stored in the Ingenuity Knowledgebase. Significant proteins > 2-ratio or < 0.5-ratio; *p* < 0.05) were uploaded into the IPA environment and core analyses were performed to identify prominent interactions and associations within each dataset, with the following amendments to the default criteria:

- Highly predicted or experimentally observed confidence levels;
- Species, mammals with stringent filtering;
- Restricted to immune cells, bone marrow cells, stem cells, immune cell lines, leukemia cell lines, lymphoma cell lines and other cells not otherwise specified.

A large comparative analysis was performed to identify common pathways, nodes and/or regulators between the different drug treatments. Using the Path Explorer tool and predicted up-stream regulators, we built a representative interaction network in the IPA environment by expanding known direct and indirect connections between significant targets from all three datasets. Molecules representing HSP-90 and the DNA-strand break marker, H2AX (gene name, *H2AFX*) were added and connected to the network using the IPA knowledgebase. All datasets were then overlaid in turn and activation states were predicted.

### Western blot analyses

Triplicate MEC1 protein samples (15 μg) from each condition were separated by 10% SDS-PAGE, and transferred to a PVDF membrane (Immun-Blot™, Bio-Rad Laboratories, Hercules, CA, USA). After blocking with 5% skim milk in TBST (25 mM Tris/HCl, 137 mM NaCl, 2.7 mM KCl and 0.1% (v/v) Tween-20, pH 7.6), the membranes were incubated in TBST (4°C, 16 h) with rabbit monoclonal antibodies against BRCA1, NCL, NFKB2 p100/p52 or MYC (Cell Signalling Technology, Danvers, USA) or mouse monoclonal antibody against CCND1 (Cell Signalling Technology) and actin (Abcam, Cambridge, USA), followed by incubation (1 h, room temperature) with horse radish peroxidase (HRP)-conjugated secondary antibodies: goat-anti-mouse-HRP (Santa Cruz Biotechnology Inc., Santa Cruz, CA, USA) or donkey-anti-rabbit-HRP (Abcam, Cambridge, UK). Proteins were visualized using Rapid Step ECL Reagent (Merck, Kilsyth, Victoria, Australia) and ECL chemiluminescence film (GE Healthcare, Little Chalfont, Buckinghamshire, UK). Films were scanned on a Molecular Imager GS-800™ densitometer (Bio-Rad, Hercules, CA, USA). Bands were quantified using ImageQuantTL density analysis software (GE Healthcare, Little Chalfont, Buckinghamshire, UK) and two-tailed homoscedastic student *t*-tests were performed on log_2_-transformed, actin-normalized ratios to the control samples.

### Flow cytometry

Levels of γH2AX were determined in triplicate samples of MEC1, MEC2 and U266 drug-treated and control cells using a FACScalibur flow cytometer (FACSCalibur; Becton Dickinson, Franklin Lakes, NJ, USA) with a 488 nm argon laser, running CellQuest Pro software version 5.2. Cells were labelled with γH2AX antibody conjugated to Alexa-488 (Abcam, Cambridge, MA, USA) using standard procedures. Flow cytometry with fluorescence detection used logarithmic amplification and biological replicates were averaged.

## CONCLUSIONS

Quantitative label-free shotgun LC-MS/MS, employing spectral counting with NSAFs, was used to investigate changes in protein levels in human MEC1 CLL cells treated with the Hsp90 inhibitor SNX-7081, the purine analog 2-FaraA, and dual treatment with both drugs. The data obtained are consistent with the following mechanism of synergy between SNX-7081 and 2-FaraA: 2-FaraA induces DNA damage, SNX-7081 down-regulates DNA repair proteins with accumulation of damaged cells that undergo apoptosis that may be mediated by a SNX-7081 induced loss of MYC and NF*k*B2 p100 accumulation. Induction of the DNA damage marker, γH2AX, indicates that a similar mechanism may operate in other p53-mutated human B-lymphoid cancers, such as the cell lines MEC2 (CLL) and U266 (multiple myeloma). These results provide valuable insight into the synergistic mechanism between SNX-7081 and 2-FaraA that may provide an alternative treatment for CLL patients with p53 mutations, where options are currently limited [10]. This drug combination reduces the effective dose of the Hsp90 inhibitor and could, therefore, overcome the toxicity encountered in clinical trials of Hsp90 inhibitors.

## SUPPLEMENTARY FIGURES AND TABLE





## Abbreviations

2-FaraA: fludarabine nucleoside
AARS: amino acyl tRNA synthetases
ACN: acetonitrile
BASC: BRCA1-associated genome surveillance complex
BRCA1: breast cancer gene 1
CCND1: cyclin D1
CLL: chronic lymphocytic leukemia
DNA-PK: DNA-dependent serine/threonine-protein kinase
DSB: double strand breaks
DTT: dithiothreitol
FAF2: fas-associated factor 2
FdATP: fludarabine triphosphate
FDR: false discovery rate
HR: homologous recombination
HRP: horse radish peroxidase
Hsp90: heat shock protein 90
IPA: Ingenuity® Pathway Analysis
LC-MS/MS: liquid chromatography tandem mass spectrometry
MMR: mismatch repair system
MYC: myelocytomatosis oncogene cellular homolog
NCL: nucleolin
NF*k*B2: nuclear factor-kappa-B p100 subunit
NHEJ: non-homologous end joining
NSAF: normalized spectral abundance factor
PML: promyelocytic leukemia protein
RFC: replication factor C
XRCC5: X-ray repair cross-complementing protein 5
YARS: tyrosyl-tRNA synthetase

## References

[1] Zenz T, Krober A, Scherer K, Habe S, Buhler A, Benner A, Denzel T, Winkler D, Edelmann J, Schwanen C, Dohner H, Stilgenbauer S (2008). Monoallelic TP53 inactivation is associated with poor prognosis in chronic lymphocytic leukemia: results from a detailed genetic characterization with long-term follow-up. Blood.

[2] Mactier S, Henrich S, Che Y, Kohnke P.L, Christopherson R.I (2011). Comprehensive proteomic analysis of the effects of purine analogs on human Raji B-cell lymphoma. J Proteome Res.

[3] Amundson S.A, Myers T.G, Fornace A.J (1998). Roles for p53 in growth arrest and apoptosis: putting on the brakes after genotoxic stress. Oncogene.

[4] Bocangel D, Sengupta S, Mitra S, Bhakat K.K (2009). p53-Mediated down-regulation of the human DNA repair gene O6-methylguanine-DNA methyltransferase (MGMT) via interaction with Sp1 transcription factor. Anticancer Res.

[5] Adimoolam S, Ford J.M (2003). p53 and regulation of DNA damage recognition during nucleotide excision repair. DNA Repair (Amst).

[6] Donehower L.A (2009). Longevity regulation in flies: a role for p53. Aging (Albany NY).

[7] Wattel E, Preudhomme C, Hecquet B, Vanrumbeke M, Quesnel B, Dervite I, Morel P, Fenaux P (1994). p53 mutations are associated with resistance to chemotherapy and short survival in hematologic malignancies. Blood.

[8] Silber R, Degar B, Costin D, Newcomb E.W, Mani M, Rosenberg C.R, Morse L, Drygas J.C, Canellakis Z.N, Potmesil M (1994). Chemosensitivity of lymphocytes from patients with B-cell chronic lymphocytic leukemia to chlorambucil, fludarabine, camptothecin analogs. Blood.

[9] Enoch T, Norbury C (1995). Cellular responses to DNA damage: cell-cycle checkpoints, apoptosis and the roles of p53 and ATM. Trends Biochem Sci.

[10] Best O.G, Che Y, Singh N, Forsyth C, Christopherson R.I, Mulligan S.P (2012). The Hsp90 inhibitor SNX-7081 synergizes with and restores sensitivity to fludarabine in chronic lymphocytic leukemia cells with lesions in the TP53 pathway: a potential treatment strategy for fludarabine refractory disease. Leuk Lymphoma.

[11] Goetz M.P, Toft D.O, Ames M.M, Erlichman C (2003). The Hsp90 chaperone complex as a novel target for cancer therapy. Ann Oncol.

[12] Brown M.A, Zhu L, Schmidt C, Tucker P.W (2007). Hsp90—from signal transduction to cell transformation. Biochem Biophys Res Commun.

[13] Maloney A, Workman P (2002). HSP90 as a new therapeutic target for cancer therapy: the story unfolds. Expert Opin Biol Ther.

[14] Kim Y.S, Alarcon S.V, Lee S, Lee M.J, Giaccone G, Neckers L, Trepel J.B (2009). Update on Hsp90 inhibitors in clinical trial. Curr Top Med Chem.

[15] Best O.G, Singh N, Forsyth C, Mulligan S.P (2010). The novel Hsp-90 inhibitor SNX7081 is significantly more potent than 17-AAG against primary CLL cells and a range of haematological cell lines, irrespective of lesions in the TP53 pathway. Br J Haematol.

[16] Che Y, Best O.G, Zong L, Kaufman K.L, Mactier S, Raftery M.J, Graves L.M, Mulligan S.P, Christopherson R.I (2013). The Hsp90 inhibitor SNX-7081, dysregulates proteins involved with DNA repair and replication and the cell cycle in human chronic lymphocytic leukemia (CLL) cells. J Proteome Res.

[17] Rajan A, Kelly R.J, Trepel J.B, Kim Y.S, Alarcon S.V, Kummar S, Gutierrez M, Crandon S, Zein W.M, Jain L, Mannargudi B, Figg W.D, Houk B.E (2011). A phase I study of PF-04929113 (SNX-5422), an orally bioavailable heat shock protein 90 inhibitor, in patients with refractory solid tumor malignancies and lymphomas. Clin Cancer Res.

[18] Henrich S, Christopherson R.I (2008). Multiple forms of nuclear p53 formed in human Raji and MEC1 cells treated with fludarabine. Leukemia.

[19] Mah L.J, El-Osta A, Karagiannis T.C (2010). gammaH2AX: a sensitive molecular marker of DNA damage and repair. Leukemia.

[20] Henrich S, Mactier S, Best G, Mulligan S.P, Crossett B, Christopherson R.I (2011). Fludarabine nucleoside modulates nuclear “survival and death” proteins in resistant chronic lymphocytic leukemia cells. Nucleosides Nucleotides Nucleic Acids.

[21] Moussay E, Palissot V, Vallar L, Poirel H.A, Wenner T, El Khoury V, Aouali N, Van Moer K, Leners B, Bernardin F, Muller A, Cornillet-Lefebvre P, Delmer A (2010). Determination of genes and microRNAs involved in the resistance to fludarabine *in vivo* in chronic lymphocytic leukemia. Mol Cancer.

[22] Jitschin R, Braun M, Qorraj M, Saul D, Le Blanc K, Zenz T, Mougiakakos D (2015). Stromal cell-mediated glycolytic switch in CLL cells involves Notch-c-Myc signaling. Blood.

[23] Hoffman B, Liebermann D.A (1998). The proto-oncogene c-myc and apoptosis. Oncogene.

[24] Hsu B, Marin M.C, el-Naggar A.K, Stephens L.C, Brisbay S, McDonnell T.J (1995). Evidence that c-myc mediated apoptosis does not require wild-type p53 during lymphomagenesis. Oncogene.

[25] Harte M.T, Gorski J.J, Savage K.I, Purcell J.W, Barros E.M, Burn P.M, McFarlane C, Mullan P.B, Kennedy R.D, Perkins N.D, Harkin D.P (2014). NF-kappaB is a critical mediator of BRCA1-induced chemoresistance. Oncogene.

[26] Matsuzawa A, Kanno S, Nakayama M, Mochiduki H, Wei L, Shimaoka T, Furukawa Y, Kato K, Shibata S, Yasui A, Ishioka C, Chiba N (2014). The BRCA1/BARD1-interacting protein OLA1 functions in centrosome regulation. Mol Cell.

[27] Sankaran S, Starita L.M, Groen A.C, Ko M.J, Parvin J.D (2005). Centrosomal microtubule nucleation activity is inhibited by BRCA1-dependent ubiquitination. Mol Cell Biol.

[28] Greenberg R.A, Sobhian B, Pathania S, Cantor S.B, Nakatani Y, Livingston D.M (2006). Multifactorial contributions to an acute DNA damage response by BRCA1/BARD1-containing complexes. Genes Dev.

[29] Mullan P.B, Gorski J.J, Harkin D.P (2006). BRCA1--a good predictive marker of drug sensitivity in breast cancer treatment?. Biochim Biophys Acta.

[30] Quinn J.E, Kennedy R.D, Mullan P.B, Gilmore P.M, Carty M, Johnston P.G, Harkin D.P (2003). BRCA1 functions as a differential modulator of chemotherapy-induced apoptosis. Cancer Res.

[31] Treszezamsky A.D, Kachnic L.A, Feng Z, Zhang J, Tokadjian C, Powell S.N (2007). BRCA1- and BRCA2-deficient cells are sensitive to etoposide-induced DNA double-strand breaks via topoisomerase II. Cancer Res.

[32] Quinn J.E, James C.R, Stewart G.E, Mulligan J.M, White P, Chang G.K, Mullan P.B, Johnston P.G, Wilson R.H, Harkin D.P (2007). BRCA1 mRNA expression levels predict for overall survival in ovarian cancer after chemotherapy. Clin Cancer Res.

[33] Vlachostergios P.J, Patrikidou A, Daliani D.D, Papandreou C.N (2009). The ubiquitin-proteasome system in cancer, a major player in DNA repair. Part 1: post-translational regulation. J Cell Mol Med.

[34] Motegi A, Murakawa Y, Takeda S (2009). The vital link between the ubiquitin-proteasome pathway and DNA repair: impact on cancer therapy. Cancer Lett.

[35] Mallawaaratchy D.M, Mactier S, Kaufman K.L, Blomfield K, Christopherson R.I (2012). The phosphoinositide 3-kinase inhibitor LY294002, decreases aminoacyl-tRNA synthetases, chaperones and glycolytic enzymes in human HT-29 colorectal cancer cells. J Proteomics.

[36] Wei N, Shi Y, Truong L.N, Fisch K.M, Xu T, Gardiner E, Fu G, Hsu Y.S, Kishi S, Su A.I, Wu X, Yang X.L (2014). Oxidative stress diverts tRNA synthetase to nucleus for protection against DNA damage. Mol Cell.

[37] Polo S.E, Jackson S.P (2011). Dynamics of DNA damage response proteins at DNA breaks: a focus on protein modifications. Genes Dev.

[38] Reddy Y.V, Ding Q, Lees-Miller S.P, Meek K, Ramsden D.A (2004). Non-homologous end joining requires that the DNA-PK complex undergo an autophosphorylation-dependent rearrangement at DNA ends. J Biol Chem.

[39] Tuteja N, Tuteja R, Ochem A, Taneja P, Huang N.W, Simoncsits A, Susic S, Rahman K, Marusic L, Chen J (1994). Human DNA helicase II: a novel DNA unwinding enzyme identified as the Ku autoantigen. EMBO J.

[40] Chung U, Igarashi T, Nishishita T, Iwanari H, Iwamatsu A, Suwa A, Mimori T, Hata K, Ebisu S, Ogata E, Fujita T, Okazaki T (1996). The interaction between Ku antigen and REF1 protein mediates negative gene regulation by extracellular calcium. J Biol Chem.

[41] Willis D.M, Loewy A.P, Charlton-Kachigian N, Shao J.S, Ornitz D.M, Towler D.A (2002). Regulation of osteocalcin gene expression by a novel Ku antigen transcription factor complex. J Biol Chem.

[42] Clark A.B, Valle F, Drotschmann K, Gary R.K, Kunkel T.A (2000). Functional interaction of proliferating cell nuclear antigen with MSH2-MSH6 and MSH2-MSH3 complexes. J Biol Chem.

[43] Ohta S, Shiomi Y, Sugimoto K, Obuse C, Tsurimoto T (2002). A proteomics approach to identify proliferating cell nuclear antigen (PCNA)-binding proteins in human cell lysates. Identification of the human CHL12/RFCs2-5 complex as a novel PCNA-binding protein. J Biol Chem.

[44] Kleczkowska H.E, Marra G, Lettieri T, Jiricny J (2001). hMSH3 and hMSH6 interact with PCNA and colocalize with it to replication foci. Genes Dev.

[45] Wang Y, Cortez D, Yazdi P, Neff N, Elledge S.J, Qin J (2000). BASC, a super complex of BRCA1-associated proteins involved in the recognition and repair of aberrant DNA structures. Genes Dev.

[46] Lindsey-Boltz L.A, Bermudez V.P, Hurwitz J, Sancar A (2001). Purification and characterization of human DNA damage checkpoint Rad complexes. Proc Natl Acad Sci U S A.

[47] Li Y, Araki H (2013). Loading and activation of DNA replicative helicases: the key step of initiation of DNA replication. Genes Cells.

[48] Dellaire G, Bazett-Jones D.P (2004). PML nuclear bodies: dynamic sensors of DNA damage and cellular stress. Bioessays.

[49] Cairo S, De Falco F, Pizzo M, Salomoni P, Pandolfi P.P, Meroni G (2005). PML interacts with Myc, Myc target gene expression is altered in PML-null fibroblasts. Oncogene.

[50] Wang J.C (2002). Cellular roles of DNA topoisomerases: a molecular perspective. Nat Rev Mol Cell Biol.

[51] Rogakou E.P, Pilch D.R, Orr A.H, Ivanova V.S, Bonner W.M (1998). DNA double-stranded breaks induce histone H2AX phosphorylation on serine 139. J Biol Chem.

[52] Ku S, Lasorsa E, Adelaiye R, Ramakrishnan S, Ellis L, Pili R (2014). Inhibition of Hsp90 augments docetaxel therapy in castrate resistant prostate cancer. PLoS One.

[53] Regan P.L, Jacobs J, Wang G, Torres J, Edo R, Friedmann J, Tang X.X (2011). Hsp90 inhibition increases p53 expression and destabilizes MYCN and MYC in neuroblastoma. Int J Oncol.

[54] Cowling V.H, Cole M.D (2010). Myc Regulation of mRNA Cap Methylation. Genes Cancer.

[55] Keller U, Huber J, Nilsson J.A, Fallahi M, Hall M.A, Peschel C, Cleveland J.L (2010). Myc suppression of Nfkb2 accelerates lymphomagenesis. BMC Cancer.

[56] Jing H, Lee S (2014). NF-kappaB in cellular senescence and cancer treatment. Mol Cells.

[57] Bernal-Mizrachi L, Edwards S.K, Ratner L (2005). Accumulation of NFkB1 (p105) and NFkB2 (p100) Is Essential for Apoptosis Induced by Proteasome Inhibition in a Lymphoma Model. Blood.

[58] Walsby E, Pearce L, Burnett A.K, Fegan C, Pepper C (2012). The Hsp90 inhibitor NVP-AUY922-AG inhibits NF-kappaB signaling, overcomes microenvironmental cytoprotection and is highly synergistic with fludarabine in primary CLL cells. Oncotarget.

[59] Xu J.Y, Lu S, Xu X.Y, Hu S.L, Li B, Qi R.X, Chen L, Chang J.Y (2015). Knocking Down Nucleolin Expression Enhances the Radiosensitivity of Non-Small Cell Lung Cancer by Influencing DNA-PKcs Activity. Asian Pac J Cancer Prev.

[60] Mi Y, Thomas S.D, Xu X, Casson L.K, Miller D.M, Bates P.J (2003). Apoptosis in leukemia cells is accompanied by alterations in the levels and localization of nucleolin. J Biol Chem.

[61] Wise J.F, Berkova Z, Mathur R, Zhu H, Braun F.K, Tao R.H, Sabichi A.L, Ao X, Maeng H, Samaniego F (2013). Nucleolin inhibits Fas ligand binding and suppresses Fas-mediated apoptosis *in vivo* via a surface nucleolin-Fas complex. Blood.

[62] Wang S.A, Li H.Y, Hsu T.I, Chen S.H, Wu C.J, Chang W.C, Hung J.J (2011). Heat shock protein 90 stabilizes nucleolin to increase mRNA stability in mitosis. J Biol Chem.

[63] Stacchini A, Aragno M, Vallario A, Alfarano A, Circosta P, Gottardi D, Faldella A, Rege-Cambrin G, Thunberg U, Nilsson K, Caligaris-Cappio F (1999). MEC1 and MEC2: two new cell lines derived from B-chronic lymphocytic leukaemia in prolymphocytoid transformation. Leuk Res.

[64] Gong B, Almasan A (1999). Differential upregulation of p53-responsive genes by genotoxic stress in hematopoietic cells containing wild-type and mutant p53. Gene Expr.

[65] Mirzaei M, Soltani N, Sarhadi E, Pascovici D, Keighley T, Salekdeh G.H, Haynes P.A, Atwell B.J (2012). Shotgun proteomic analysis of long-distance drought signaling in rice roots. J Proteome Res.

[66] Zybailov B, Mosley A.L, Sardiu M.E, Coleman M.K, Florens L, Washburn M.P (2006). Statistical analysis of membrane proteome expression changes in *Saccharomyces cerevisiae*. J. Prot. Res.

[67] Neilson K.A, Keighley T, Pascovici D, Cooke B, Haynes P.A (2013). Label-free quantitative shotgun proteomics using normalized spectral abundance factors. Methods Mol Biol.

[68] McDonald J.H (2009). Handbook of Biological Statistics.

