# Supplementary material for: The Hsp90 inhibitor SNX-7081 is synergistic with fludarabine nucleoside via DNA damage and repair mechanisms in human, p53-negative chronic lymphocytic leukemia
Source: Oncotarget. 2015 Nov 6;6(38):40981–97. doi: 10.18632/oncotarget.5715 (PMC4747384; doi:10.18632/oncotarget.5715)
Supplement: Supplementary file 1 [file oncotarget-06-40981-s001.pdf]

SUPPLEMENTARY FIGURES AND TABLE

A.

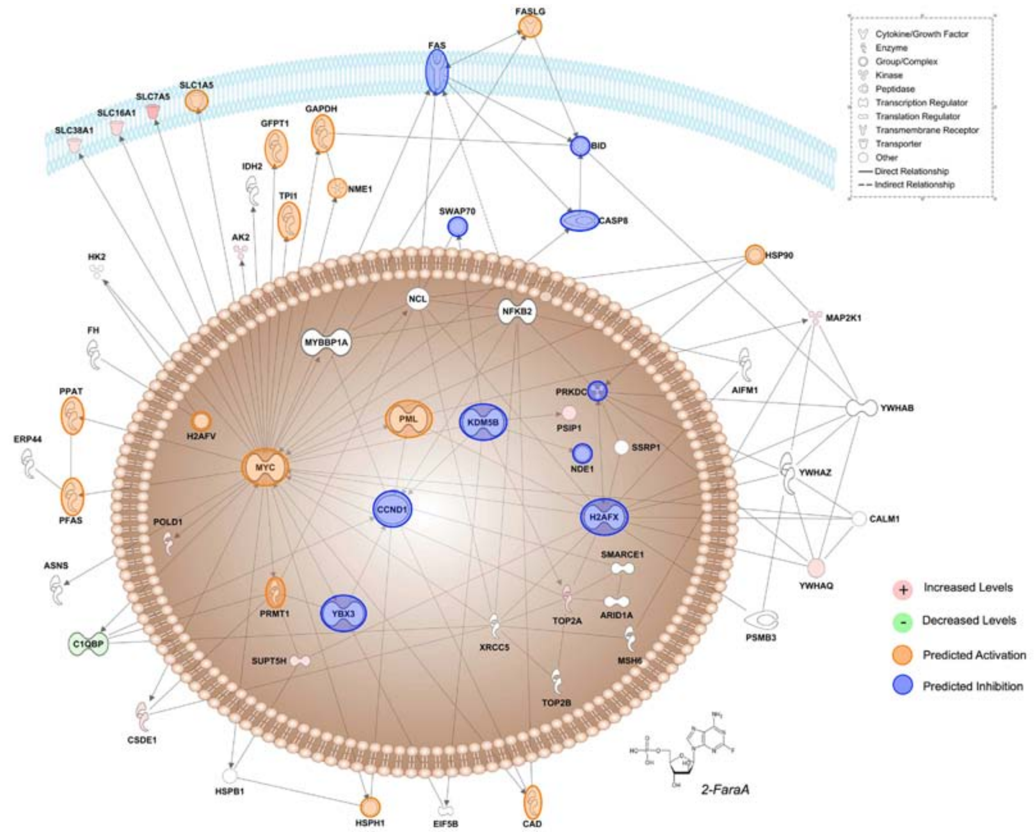

(*continued*)

B.

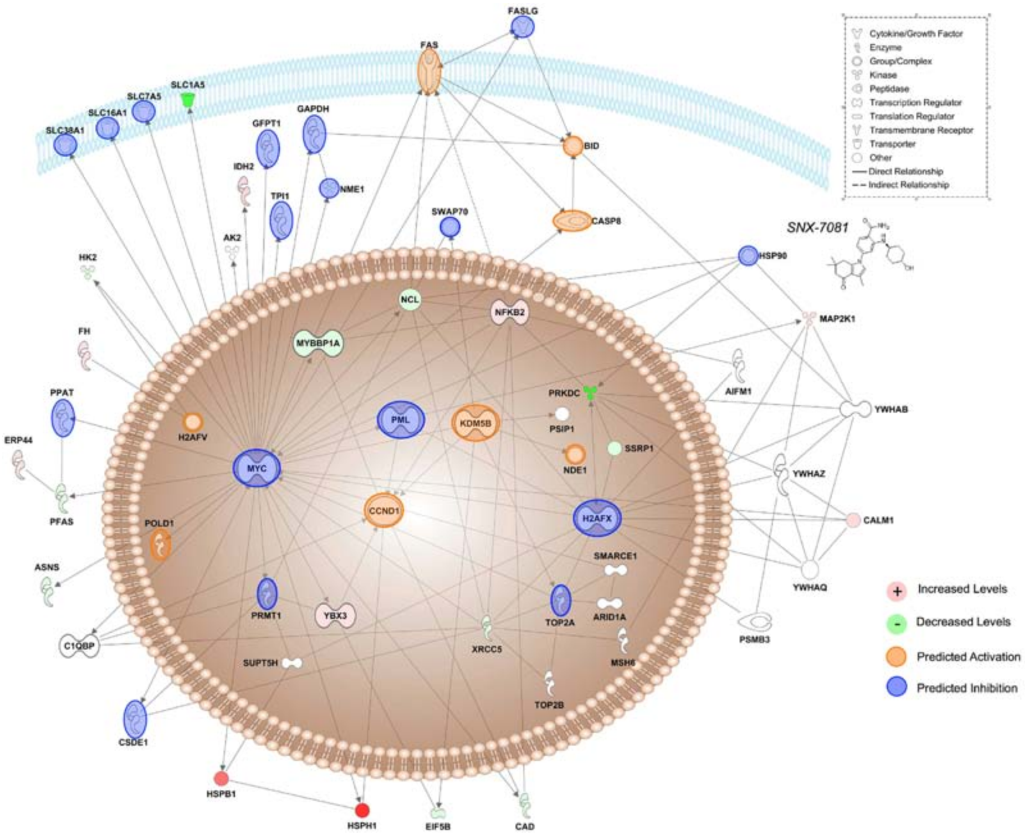
FASLG
FAS
SLC1A5
SLC7A5
SLC16A1
SLC38A1
GFPT1
GAPDH
BID
IDH2
TPI1
NME1
AK2
SWAP70
CASP8
SNX-7081
HSP90
HK2
NCL
NFKB2
MAP2K1
FH
MYBBP1A
AIFM1
PPAT
PRKDC
YWHAB
PML
KDM5B
PSIP1
ERP44
H2AFV
NDE1
SSRP1
MYC
YWHAZ
PFAS
CCND1
H2AFX
CALM1
POLD1
SMARCE1
ASNS
YWHAQ
PRMT1
YBX3
TOP2A
ARID1A
PSMB3
C1QBP
XRCC5
MSH6
SUPT5H
TOP2B
CSDE1
HSPB1
HSPH1
EIF5B
CAD
Cytokine/Growth Factor
Enzyme
Group/Complex
Kinase
Peptidase
Transcription Regulator
Translation Regulator
Transmembrane Receptor
Transporter
Other
Direct Relationship
Indirect Relationship
Increased Levels
Decreased Levels
Predicted Activation
Predicted Inhibition

(*continued*)

C.

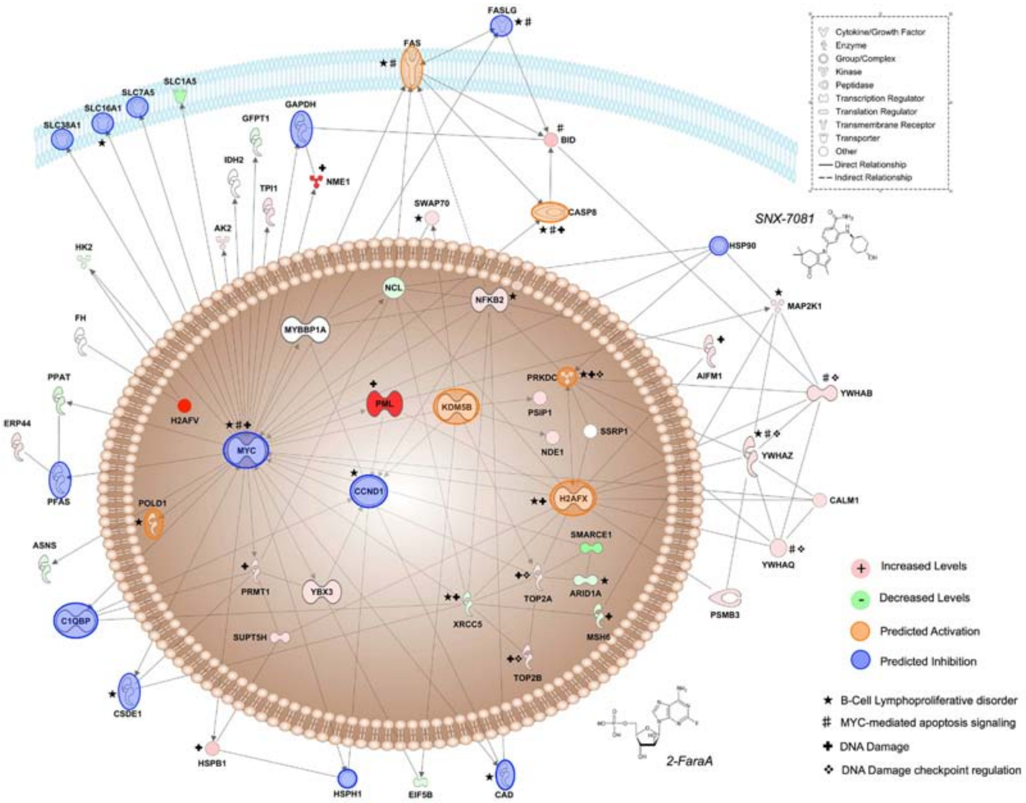

Supplementary Figure S1 (*continued*): Using IPA software, an interaction network comprising 58 molecules (indicated by their gene names) was generated and overlaid with A. protein abundance changes following 2-FaraA treatment, B. SNX-7081 and C. dual treatment. Hsp90 and H2AX molecules were added to the system to test their predicted activation states; as expected Hsp90 is inhibited after both treatment conditions with the SNX-7081 molecule and H2AX is activated after dual drug treatment, supporting the observation of increased levels of γH2AX (Figure 5). Proteins with increased levels (>2-fold) after drug treatment are in red and proteins with decreased levels (<0.5-ratio) are in green, with higher ratio changes signified by more saturated colours. Predicted activations are in orange, and predicted inhibitions are in blue. Molecules previously linked to B-cell lymphoproliferative disorders, MYC-mediated apoptosis signaling, DNA damage and DNA damage checkpoint regulation are annotated.

Supplementary Table S1: Master list of significant protein abundance changes across all treatment conditions
